# Ductility of the Tensile Zone in Bent Wooden Beams Strengthened with CFRP Materials

**DOI:** 10.3390/ma13235451

**Published:** 2020-11-30

**Authors:** Agnieszka Wdowiak-Postulak, Janusz Brol

**Affiliations:** 1 Faculty of Civil Engineering and Architecture, Kielce University of Technology, 25-314 Kielce, Poland; 2 Faculty of Civil Engineering, Silesian University of Technology, 44-100 Gliwice, Poland; janusz.brol@polsl.pl

**Keywords:** glued laminated beams, solid wooden beams, CFRP materials, strengthening, bending strength

## Abstract

This article presents experimental results from the bending of technical-scale models of beams reinforced in the tension zone with CFRP (Carbon Fiber Reinforced Polymers) materials, with a focus on the benefits resulting from the increased ductility in the tension zone of these beams. In experimental tests, the mechanical properties of reinforced beams were compared with unreinforced beams in terms of the maximum load, deflection, images of damage, stiffness, and distribution of deformation. The results showed that the proposed reinforcement solution was advantageous due to its strength and stiffness, and the safety of the structure. Based on this analysis, it was concluded that the reinforcement of wood with CFRP materials has a positive effect on the behavior and safety of structures. Also, a method of analytical checking of strengthened beams with small cross-sections was presented in the article.

## 1. Introduction

Despite the development of steel and concrete structures, wooden elements are still used in modern constructions. The advantages of wood include its lightness, relatively high durability, ease of assembly, attractive appearance and, most importantly, the lack of emission of environmental pollutants. These are only a few of the features that affect the use of timber in construction. Unfortunately, wood also has several disadvantages, including its variability in terms of strength and stiffness, which is caused by the presence of natural defects and fluctuations in growing conditions and makes it difficult to determine the behavior of wooden elements under different loads [1,2]. These problems can be solved through the use of wood-based products such as laminated timber, in which smaller sections of solid wood are glued together, thus enabling the production of wooden elements in which defects are dispersed and giving a final product with more uniform properties and unlimited dimensions [1,3].

In the case of solid wood (and, to a lesser extent, glued wood), there are natural defects in the structure, such as knots or twisted fibers, that have a negative impact, particularly in the tensile zone, as they cause non-ductility in the stretched zone in both bent and stretched elements. This non-ductility in the compressed zones of the wooden sections is typically a minor problem, but the damage arising during stretching is ‘‘brittle’’; it can be said that the wood does not show plasticity under tensile loads, while in the compressed zone it is plastic. We can therefore counteract the structural non-ductility of wood caused by local defects by strengthening the tension zone with another material.

All of the effective wood strengthening techniques that currently exist aim to reduce the cross-sectional size of the beams, and to use either lower strength species of wood or wood of a lower strength class, thus enabling a wider use of the available wood resources. It is also possible to increase the load-bearing capacity of the existing wooden elements to allow them to withstand much greater loads, thus creating savings in terms of the money and material needed to replace the existing structure. In the past, laminated beams with metal reinforcements (steel plates or bars, or aluminum plates) have been tested for bending. This is a simple and effective method, but has certain disadvantages, such as difficulties in transport and assembly, huge maintenance costs, corrosion, and the bending of aluminum plates. Over the last decade, there has been increased interest in research into the reinforcement of wood with fibrous composite materials, due to their wide availability and lower cost. It should be noted here that in textbooks on composites, wood is often mentioned as an example of a natural composite, so we can say that “a composite is strengthened with a composite”.

The three most common types of fibers used in structural elements are glass, carbon, and aramid. More recently, basalt fibers have also been used [4,5]. Their advantages include high stiffness and tensile strength, low weight, easy assembly, high strength (no corrosion), electromagnetic permeability, and virtually unlimited availability in terms of geometry and size. Moreover, these materials can be factory-produced with very close tolerances for their mechanical properties [1].

To improve the mechanical properties of wood, experimental tests have been carried out that have involved combining wood with other materials such as FRPs. Typically, the FRP materials used for this reinforcement have been in the form of fabrics or bars.

Experimental studies on materials reinforced with synthetic fibers (such as glass, carbon, aramid etc.) and epoxy resins have been carried out. This research has shown that the use of these composite materials has significant structural significance, since they provide higher strength and plasticity to wooden elements, compared with the properties of unreinforced wood [6]. In particular, three beams reinforced with two basalt fiber strips showed an increase in load capacity of about 38.4%, while beams reinforced with flax fiber showed an average increase of 67.8%. In another study, Nadir et al. [7] investigated glued laminated timber reinforced with carbon fiber (CFRP) and glass fiber (GFRP) sheets, and found that there were increases in stiffness of 26.29% and 45.76%, and in bending strength of 36.91% and 40%, with GFRP reinforcement of 2.5% and 5%, respectively, compared to unreinforced beams. However, for CFRP reinforcements of 1.67% and 3.33%, the increases in stiffness were 36.19% and 64.12%, and the increases in bending strength were 45.86% and 50.62%, respectively. Li et al. [8] investigated the use of a CFRP sheet in the tension zone for two different types of timber beams. From these tests, it was found that an increase in the number of CFRP sheets decreased the displacement of the reinforced beams. It was also shown that the increases in bending strength for Cunninghamia lanceolata with one, two and three layers of CFRP sheets were 39%, 44% and 61%, respectively, while for Tsuga chinensis with one, two, and three CFRP sheets, these increases were 44%, 55% and 58%, respectively. Glišović et al. [1] examined glued laminated beams reinforced with two different layers of CFRP boards, and found that this reinforcement was effective in the tension zone. Borri and Corradi [9,10] performed tests involving the reinforcement of composite materials with CFRP and GFRP, and showed that these sheets could increase the bending stiffness. The use of three layers of carbon fiber gave an increase in bending strength of 60.3% compared to unreinforced beams. In addition, FRP composite material in the form of CFRP bars [11] was also used as a near-surface reinforcement (NSMR). The NSMR method does not increase the cross-sectional height, and protects the reinforcement from external damage. This reinforcement method increased the bending strength by an average of 49–63%. Strength tests of wooden beams reinforced with CFRPs have also been performed [12,13,14,15,16,17,18]. Triantafillou [19] carried out shear tests of glued laminated timber beams that were reinforced at the ends of their cross-sections with carbon fiber sheets, and highlighted the advantages of using carbon polymers in the production and strengthening of glued laminated beams. Studies have shown that the strengthening effect of FRP increases when the fibers are oriented in the longitudinal direction. It can be shown that there are many options for the reinforcement of wooden elements used in new constructions and in the renovation of existing structures. Wood strengthening techniques can be used to reduce the size of the beams and increase their strength, resulting in a more efficient use of wood [20,21,22,23,24,25,26]. Fiorelli and Dias [27] explored the structural behavior of GFRP- and CFRP-reinforced wood beams, and found that the bending stiffness (EI) obtained in these experimental tests was greater than the theoretical value, thus improving the safety of the structure. The increase in stiffness ranged from 15% to 29% for beams reinforced with glass and carbon fiber. Buell and Saadatmanesh [28] increased the number of fabric plies for wooden beams to compare the load-bearing capacities of these beams. It was found that the application of carbon fabric to wooden beams provided a significant increase in bending strength and a nominal increase in the stiffness of the beams. Ultimately, the bending strength of all reinforced beams increased from 40% to 53%. Alam et al. [29] reinforced cracked wooden beams with steel and FRP, and found that these reinforcements were very effective; CFRP reinforcement provided the greatest bending strength, and the researchers concluded that that addition of FRP reinforcement to timber beams could improve strength and stiffness and possibly reduce the variability in the mechanical properties of the beams compared to non-reinforced elements. The methods of analyzing the strengthening with the use of FRP in the structures are given in the works [24,30,31,32].

It should be noted that many of these studies were carried out on glued beams, and the amount of research on reinforced solid wood is still limited. Hence, in this paper, experimental studies of CFRP-reinforced glued and solid beams are presented. The aim of these tests is to determine the increases in load-bearing capacity and stiffness of solid and glued wood reinforced with CFRP fabrics. The purpose was to compare the bending strength, stiffness, and the failure images of the reinforced beams with those of the control beams. The article describes an experimental program related to the behavior of glued laminated and solid beams reinforced with carbon fiber (CFRP) during bending tests. Structural elements containing or reinforced by carbon polymers show higher strength and durability, and can be produced in various shapes. This enables use in restoration of cultural heritage in the place of their use in existing structures, they are also important in new facilities or in use of elements with reduced mechanical properties. The authors of this paper, carrying out previous studies, e.g., [5,21,25,26], with the use of various FRP (Fiber Reinforced Polymers) fibers, observed that FRP materials, and in particular CFRP, positively influenced ductility of the tensile zone of bending beams, therefore this article focuses on the impact of wood defects, in particular knots, on the behavior of beams reinforced with CFRP composite materials at the time of failure, because there is no such information in the literature on the subject. It should be remembered that in the following experimental tests, reinforcement was applied to correspond to practical scenarios of the modernization of building structures.

## 2. Materials and Methods

### 2.1. Materials

#### 2.1.1. Glued Laminated Timber

The glued and laminated timber was made of Scots pine, Pinus sylvestris L. Initially, construction timber was dried until 12% RH (Relative humidity), [33,34], and the sawn timber was then visually sorted according to PN-D-94021:2013-10 [35], with precise definition of structural and geometric features; for example, features such as knots, fiber twists, cracks, resin galls, inbarks, galls, blue stains, rot, insect holes, reaction wood (scleroderma), average grain width, density, wane, longitudinal curvature of the sides, longitudinal curvature of planes, transverse curvature in relation to width, and twist in relation to width were carefully counted, paying particular attention to knots in the wood. The coniferous construction lumber intended for the construction of beams was then divided into two classes based on its quality: KS (medium quality) and KG (inferior quality).

Glued laminated timber beams were obtained by joining four lamellas, approximately 40 mm thick, with epoxy glue (LG 815, HG 353). Bonding took place under a pressure of 0.5 to 1.0 MPa for 12 h at a temperature of about 20 °C.

**Figure 1:**
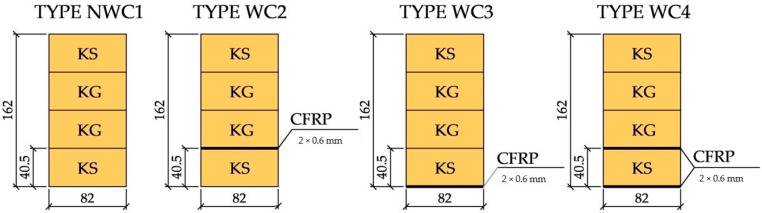
Cross-sections of the beams tested [dimensions in mm].

The final dimensions of the beams were 82 × 162 × 3650 mm^3^. Figure 1 shows the cross-sections of the beams. The average wood density was about 414.35 kg/m^3^.

#### 2.1.2. Solid Wood

Elements for the solid wood tests were also prepared from Scots pine, Pinus sylvestris L, and were also visually sorted according to PN-D-94021:2013-10 [35]. A mechanical assessment of the wood was also performed (on samples of size 20 × 20 × 300 mm^3^ taken from surplus beams) in accordance with the same standard [36] from 1999; in this approach, the strength and modulus of elasticity were determined on small samples without defects, which involved converting the strength obtained for small samples into the strength of the full-size models. The beams were classified as KS grade, which corresponds to the C24 strength class.

#### 2.1.3. CFRP

In the tests on glued wood, FRP material was used in the form of fabrics made of carbon fibers. Carbon fibers are more expensive than glass fibers, but have superior mechanical, thermal, and chemical properties. The beams were reinforced using carbon fiber of type T700S (density 1.8 g/cm^3^, tensile strength 4900 MPa, tensile modulus of elasticity 230 GPa), produced by TORAYCA (Hutton Centre Drive, Santa Ana, CA, USA).

In the tests carried out on solid wood, FRP material in the form of Sika CarboDur S512 carbon fiber tapes with a cross-section of 50 × 1.2 mm^2^ and Sika CarboDur H514 with a cross-section of 50 × 1.4 mm^2^ were glued to the bottom of the wooden beams with Sikadur-30 glue. The tensile strength of the Sika CarboDur S512 belt in our own tests was 3212 MPa, and that of the Sika CarboDur H514 belt was 1941 MPa, while the tensile moduli were 173.5 and 298.8 GPa, respectively.

#### 2.1.4. Epoxy Resin

Epoxy adhesives are commonly used for bonding composite materials. Their advantages include good gap filling, limited shrinkage during hardening, and the possibility of obtaining full curing at ambient temperature.

Epoxy glue (LG 815 + HG 353) was used to connect the lamellas and the CFRP reinforcement to the glued wood. The adhesive layer based on epoxy resin was obtained by mixing LG 815 epoxy resin (density 1.13–1.17 g/cm^3^, viscosity 1100–1300 mPa.s) with HG 353 hardener (density 0.98 g/cm^3^, viscosity 100–150 mPa.s). After mixing the resin and the hardener, the adhesive had a bending strength of 110–120 MPa and a modulus of elasticity of 2700–3300 MPa. The two parts of the epoxy adhesive (LG 815, HG 353) were mixed at a ratio of 2.5:1 by volume, as required by the product standard.

To join the CFRP tapes to the solid wood, Sikadur-30 adhesive, a ready epoxy resin, was used.

#### 2.1.5. CFRP Reinforcement Preparations

In the case of the glued timber, placing the CFRP reinforcement consisted of spreading the epoxy glue (LG 815 and HG 353) onto the surface of the beam with an approximate efficiency of 0.3 kg/m^2^, and then applying a layer of CFRP fabric. We subsequently applied a new layer of epoxy glue to the fabric so that the fibers were completely impregnated with the epoxy glue. When the reinforcement consisted of more than one layer of fiber, successive layers were created by applying epoxy glue and fabric until the required number of layers was reached. The surface of the wood was even, clean, and dry, particularly at the ends of the beam, to ensure proper anchoring. Before gluing, the surfaces of the fabrics were degreased and cleaned.

In the case of the solid wood elements, the CFRP tapes were glued to the lower sides of the beams. The surface of the wood was cleaned, the wood and CFRP tape were degreased, and the wood surface primed with Sikadur30 glue, a layer of glue was applied to the CFRP tape, and then the tape with the glue was applied to the wood surface with light, manual pressure.

### 2.2. Method

Experimental tests on the glued timber beams were carried out in the laboratory at the Department of Strength of Materials, Concrete, and Bridge Structures at the Kielce University of Technology, while experimental tests of solid wood beams were carried out in the laboratory at the Faculty of Civil Engineering at the Silesian University of Technology.

**Figure 2:**
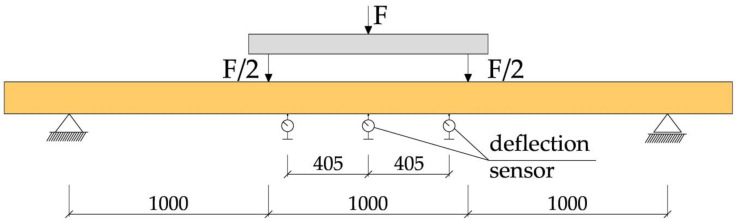
Test stand [dimensions in mm].

The test program for glued timber beams consisted of four-point bending of glued laminated beams reinforced with CFRP fabrics until failure. For each load level, the displacements and deformations were recorded, and after 20 min the data were recorded again, and the next level of force was applied. The tests were carried out in accordance with PN-EN 408+A1:2012 [36]. During the tests, the value of the loading force, the displacement of the beam in the middle of the span and at a length of 5 h (where h is the height of the beam cross-section), the deformations in the wood and the fabrics, and the value of the destructive force were recorded, and the method of failure of the tested beams was determined. The distance between the axes of the supports was 3000 mm. A schematic diagram of the test stand is shown in Figure 2.

**Figure 3:**
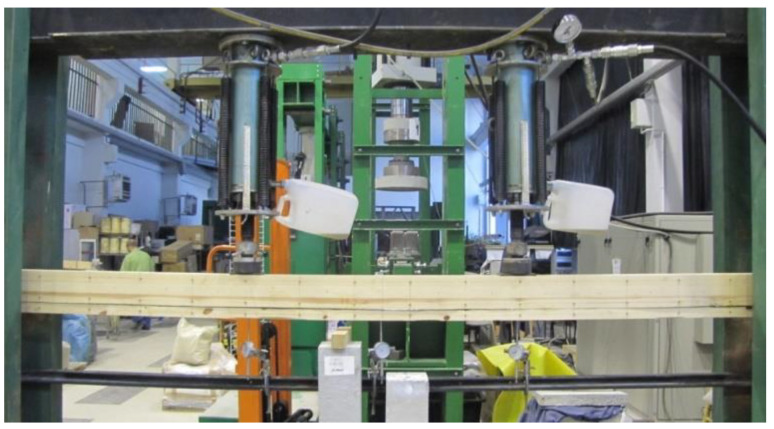
View of an exemplary model of WC2-2, reinforced along its entire length with CFRP fabric.

The displacements of the beam in the middle of the span and over a length of 5 h were measured using mechanical sensors (see Figure 2). Figure 3 shows an example of a WC2-2 beam during the experiments.

**Figure 4:**
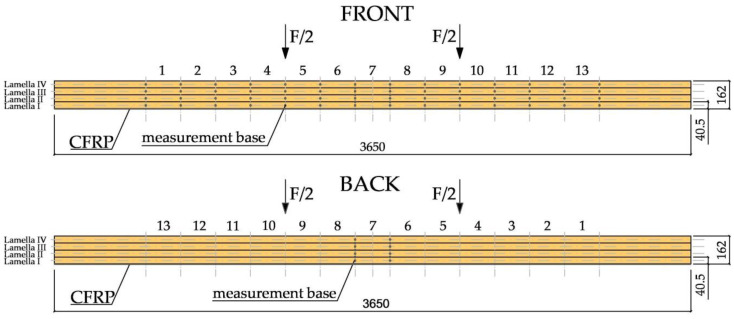
Arrangement of measuring bases along the entire length of CFRP-reinforced beams [dimensions in mm].

The deformation of the wood and CFRP fabrics was determined with the use of a “Demec” mechanical extensometer (Demec, Douglas, United Kingdom) with a defined measurement base (see Figure 4).

**Figure 5:**
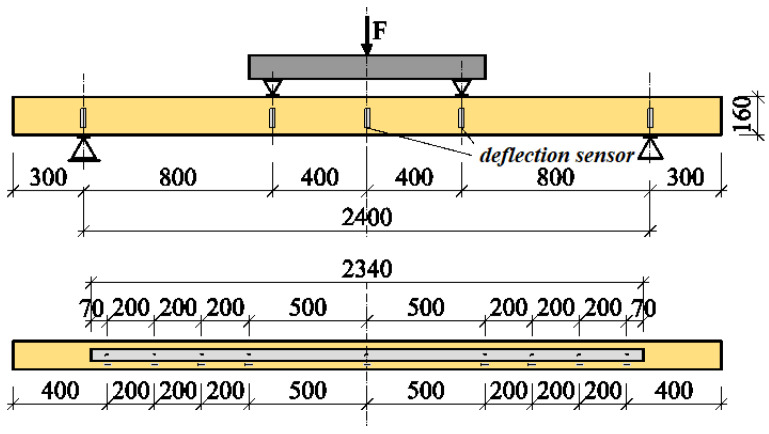
Test scheme for the ZW models, reinforced along the entire length with CFRP tape [dimensions in mm].

**Figure 6:**
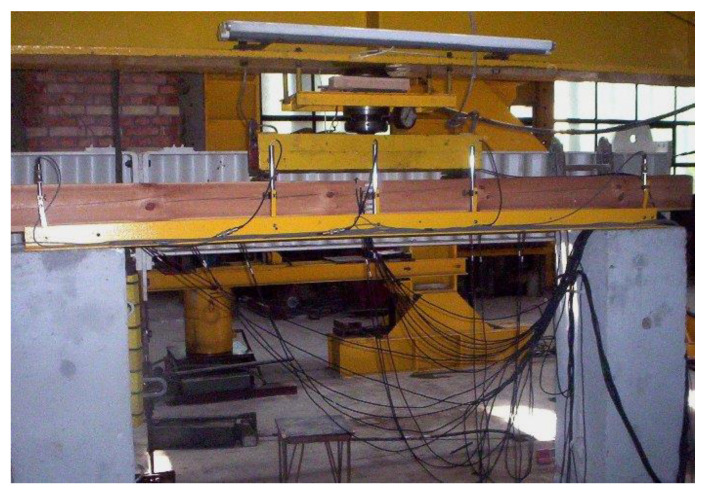
View of a ZW model, reinforced along its entire length with CFRP tape, during the test.

The test program for solid wood beams consisted of four-point bending of beams reinforced with CFRP strips until failure. The test scheme, the dimensions of the models and the arrangement of strain gauges and inductive sensors are shown in Figure 5, and a view of a reinforced beam on the test stand is shown in Figure 6. The humidity of the wood varied in all series within the range 10–12%. The load was applied in a static manner, and the model testing time was approximately 5 min +/− 120 s.

The tests were performed in three series, under immediate loading.

The ZD-2.4 series consisted of bending tests of four unreinforced wooden beams, and measurements were taken of the deflections and stresses in the wood.

The ZW-2.4S series involved bending tests of four wooden beams, reinforced with a Sika CarboDur S512 carbon fiber tape with a cross-section of 50 × 1.2 mm^2^. The percentage reinforcement was 0.313%.

The ZW-2.4H series consisted of bending tests of three wooden beams, reinforced with a Sika CarboDur H514 carbon fiber tape with a cross-section of 50 × 1.4 mm^2^. The percentage reinforcement was 0.364%. During these tests, the deflection and deformation of the wood and CFRP tape were measured and an image of the failure was taken.

The tests in the ZD-2.4 series were treated as comparative tests for the tests of the ZW-2.4S and ZW-2.4H series. The test scheme for the ZD-2.4 series models (non-reinforced beams) was identical to that used in the ZW-2.4 series. The arrangement of the inductive sensors was the same as for the ZW-2.4S and ZW-2.4H series.

## 3. Results

### 3.1. Results for Reinforced Glued Laminated Beams

The results of the load capacity for the tests of the NWC1, WC2, WC3, WC4 series beams are shown in Table 1. Taking the load capacity of the comparative elements of the NWC1 series as a reference value of 100%, it was found that the average load capacity of the WC2 series models increased by 20.9%, while for the WC3 series models, the average load capacity increased by 21.3%, while for WC4 models—28.0%.

**Figure 7:**
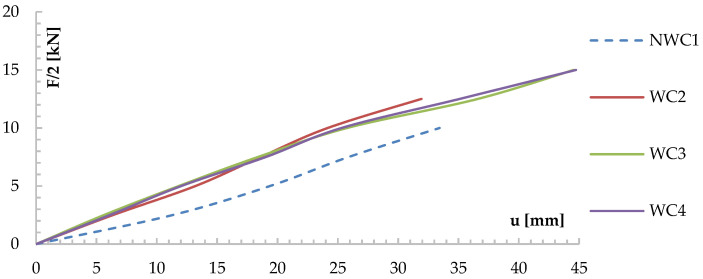
Graph of the relationship between u and F/2 for the tested beams, measured at the middle of the span.

Figure 7 shows the deflection-load curves for each test series.

The NWC1 beams were used as control beams for the WC2, WC3, and WC4 reinforced beams. The average deflection of the beams for a load F/2 of 5 kN was approximately 14.11 mm. The deflections of the beams reinforced with CFRP fabrics were much smaller than those of unreinforced beams of the NWC1 type.

**Figure 8:**
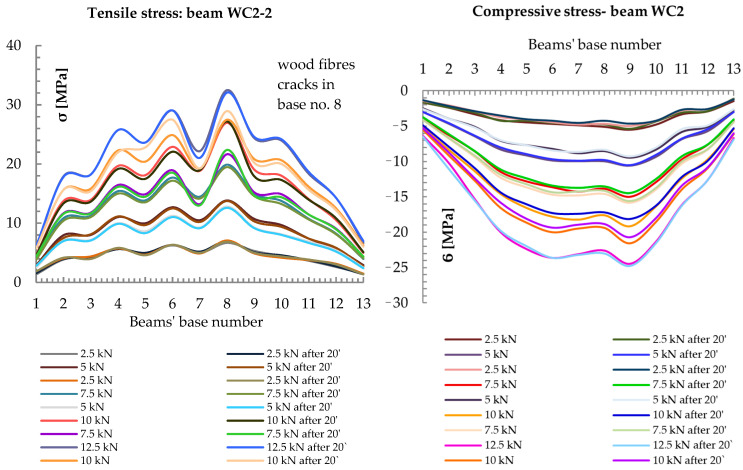
Distribution of normal stresses σ [MPa] in the tension and compression zones along the entire length of the beam reinforced with WC2-2.

Figure 8 shows a diagram of the normal stress distribution along the length of a WC2-2 reinforced beam.

The unevenness in the distribution of normal stresses was mainly due to the occurrence of defects in the wood, such as knot cracks and cracks in the wood fibers. The CFRP fabric absorbed the tensile forces, and the stresses that there increased. The CFRP fabric significantly reduced the distortion of wood fibers under tension. For a force F/2 of 10 kN (base 7), the decrease in tensile strain for the WC2 type was about 32% compared to the NWC1 type, and the decrease in compression strain was about 21%; for the WC3 type, the decrease in tensile strain was about 45% and the increase in compressive strain was about 8%; for the WC4 type, the decrease in tensile strain was about 48% and the increase in compression was about 9%.

### 3.2. Results for Reinforced Solid Wood Beams

The results of the load capacity from the tests of the ZD-2.4, ZD-2.4S, ZD-2.4H series beams are shown in Table 2. Table 2 shows the values of the breaking force and the corresponding bending moment in the models, with the standard deviation for each value. Taking the load capacity of the comparative elements of the ZD-2.4 series as a reference value of 100%, it was found that the average load capacity of the ZW-2.4S series increased by 28.1%, while for the ZW-2.4H series, the average load capacity increased by 29.1%. The values for the average strength of the reinforced models are almost identical, despite the use of two different types of tape with different modulus of elasticity and tensile strength; in the ZW-2.4S series, reinforcement consisted of tapes with a lower modulus of elasticity and higher strength (Sika CarboDur S512 tape, modulus 173.5 GPa, strength 3.21 GPa), while for the ZW-2.4H series, tapes with a higher modulus of elasticity and lower strength were used for reinforcement (Sika CarboDur H514 tape, modulus 295 GPa, strength 1.94 GPa).

**Figure 9:**
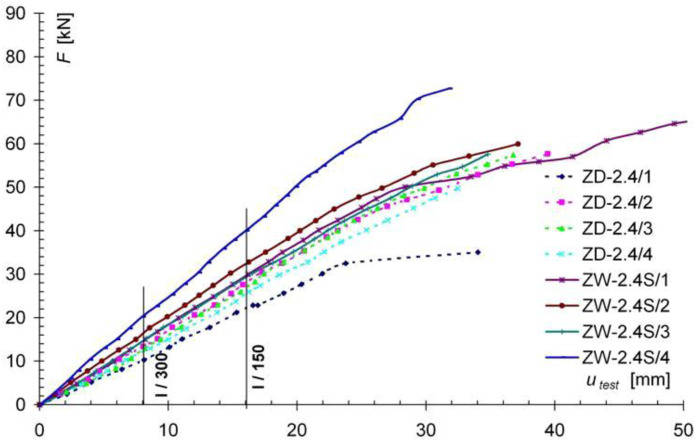
Comparison of results from the F-u_test_ for the ZD-2.4 and ZW-2.4S series models.

**Figure 10:**
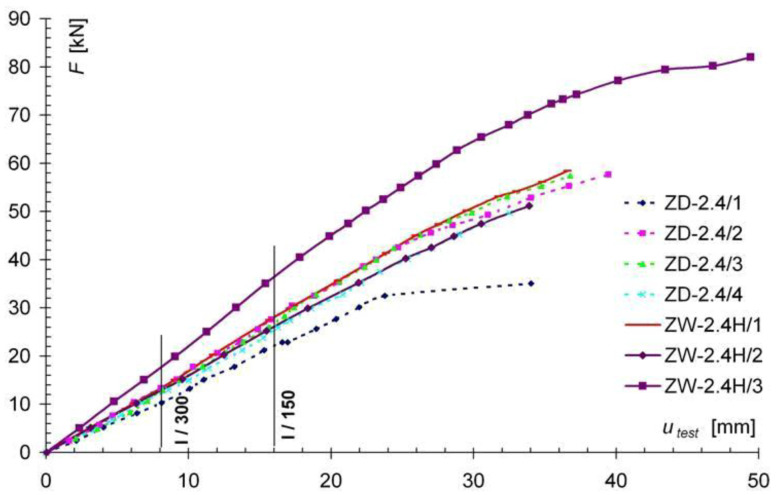
Comparison of results from the F-u_test_ for the ZD-2.4 and ZW-2.4H series models.

Figure 9 and Figure 10 show the force-deflection (F-u_test_) relationships obtained from the tests of the reinforced models of the ZD-2.4S series (Figure 9) and the ZW-2.4H series (Figure 10), and graphs of the F-u_test_ relationships for the non-reinforced models of the ZW-2.4 series as comparative models. In Figure 9 and Figure 10, the results obtained for the non-reinforced models (ZD-2.4 series) are shown with a dashed line, while the results for the reinforced models of the ZW-2.4S and ZW-2.4H series are shown with a solid line. Comparing the displacement diagrams in Figure 9, for the permissible deflection criterion of l/200, an increase in stiffness of about 20% was found for models reinforced with Sika Carbodur S512 and Sika Carbodur H514 tape (ZW-2.4S and ZW-2.4H series), compared to the non-reinforced ZD-2.4 models.

**Figure 11:**
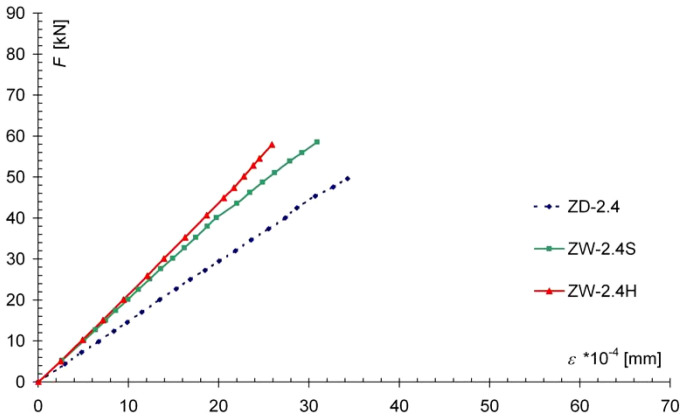
Graph of F-ε dependence for stretched marginal wood fibers in the center of the model span, showing average values.

The use of carbon fiber tape to strengthen the tensile zone of the bending elements effectively limited the deformation of the wood fibers under tension. From an analysis of the deformation of the wood in the center of the span of the beams, it was found that the tensile strains (and therefore tensile stresses) were reduced in the lower fibers of the reinforced models in relation to the non-reinforced models (Figure 11).

**Figure 12:**
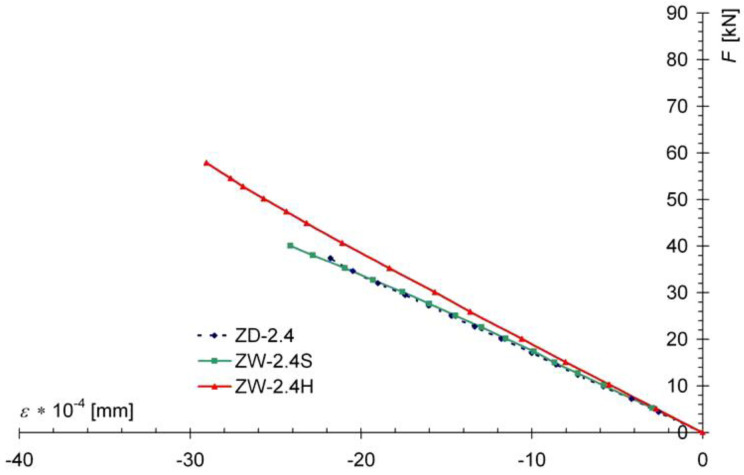
Graph of F-ε dependence in compressed marginal wood fibers in the center of the model span, showing average values.

In the case of the ZW-2.4S models, the decrease in the maximum tensile stress reached about 25%, but there was no significant change in the strains in the upper fibers (under compression), (Figure 12), while for the ZW-2.4H series models, there was a decrease in the maximum strains tensile strength of about 26%, while the deformation in the upper, compressed fibers was slightly limited. Graphs of the dependence of the average wood deformation for marginal fibers under a load F are shown in Figure 11.

**Figure 13:**
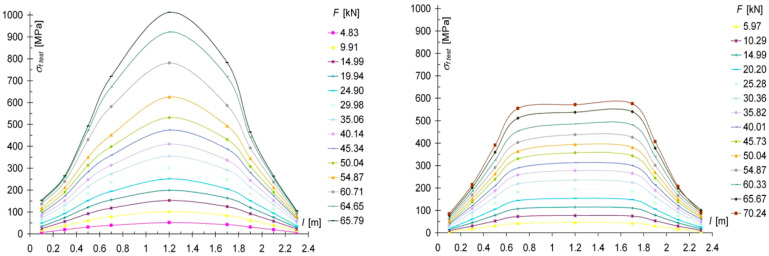
Comparison σ_f.test_—l of stress distribution in the CFRP tape for the SW-2.4S/1 (**a**) and the SW-2.4S/4 (**b**) models.

**Figure 14:**
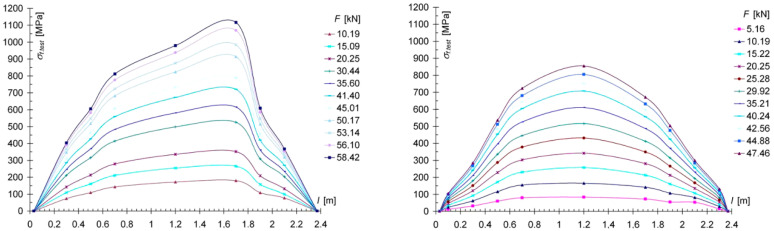
Comparison σ_f.test_—l of stress distribution in the CFRP tape for the SW-2.4H/1 (**a**) and the SW-2.4H/3 (**b**) models.

Despite the high convergence of the force-deflection (see Figure 9 and Figure 10) relationship in the center of the spans, for the individual reinforced models, the distribution of stresses in the CFRP belt along the length of the element for individual models was different, as shown in Figure 13 comparing both models of the SW-2.4S series and in Figure 14 for the two models of the SW-2.4H series. The changes in the stress distribution for the individual models were influenced by the groups of knots located near the center of the span of the SW-2.4S/1 model, and at the 1/3 of span of the SW-2.4H/1 model (under point loading force).

**Figure 15:**
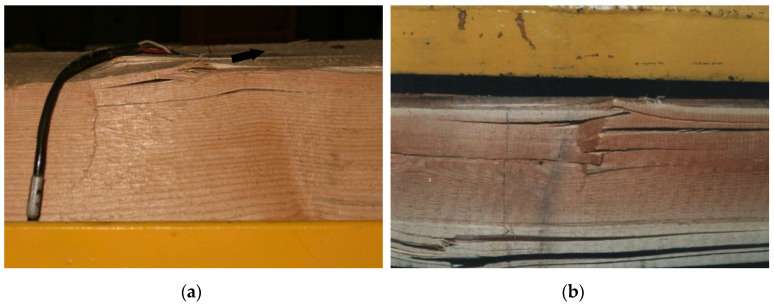
View of the wood plasticization in the compression zone in the initial phase of destruction (**a**) and at the moment of beam failure (**b**).

In the weakened places caused by defects (knots) in the wood, the tape absorbed the tensile forces and an increase in the tension in the tape could be observed. However, there was no clear decrease in the stiffness of the models. On the other hand, it was observed that the destruction of the reinforced models was gentle, and primarily occurred due to plasticization of the wood in the compressed zone of the beam (Figure 15); this mode of failure increases the safety of the structure. Figure 15 shows an image of the wood plasticization in the initial phase of failure and at the moment of beam failure.

## 4. Analytical Validation

To verify the test results, the method proposed in [30] modified by the assumption that the wood below the neutral axis does not transmit tensile stresses, and all these stresses are taken over by the CFRP tape due to its much higher longitudinal stiffness (EA) in tension than the value of longitudinal stiffness of the bottom part of the timber beam, and Eurocode 5 [37].

**Figure 16:**
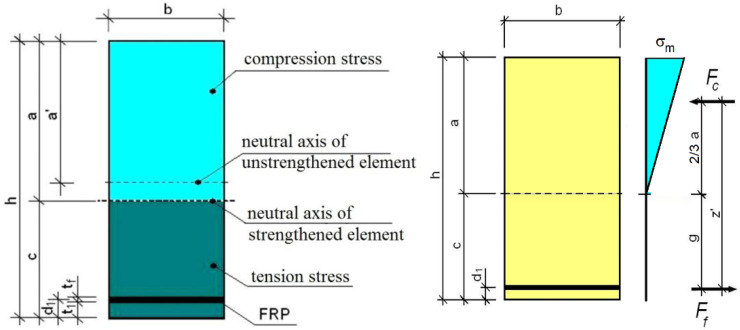
Geometry of design cross-section and design scheme used for calculations.

The location of the cross-section’s axis of inertia at bending of the strengthened timber beam was determined assuming a reduced cross-section, i.e., that the tension modulus of elasticity of the CFRP tape was reduced to the value of bending modulus of elasticity of wood using the coefficient:

(1) $$n=\frac{E_{f}}{E_{0,mean}},$$

where:

$E_{f}$—tensional modulus of elasticity of the FRP tape,

$E_{0,mean}$—mean bending modulus of elasticity of wood,

For such equivalent stiffness modulus, the height of the compression zone, in the solution with only the tension zone reinforcement, is equal to (see Figure 16):

(2) $$a=\frac{b\left(h-t_{{}_{1}}-t_{f}\right)\frac{1}{2}\left(h-t_{1}-t_{f}\right)+nb_{f}t_{f}\left(h-d_{1}\right)+bt_{1}\left(h-\frac{1}{2}t_{1}\right)}{b\left(h-t_{1}-t_{f}\right)+nb_{f}t_{f}+bt_{1}},$$

where:

h—beam’s depth,

$t_{f}$—CFRP tape’s depth,

$t_{1}$—tape “cover’s” depth,

b—beam’s width,

$b_{f}$—CFRP tape’s width,

$d_{1}$—distance from the centerline of CFRP strip to the edge of the beam.

After determining the neutral axis of the strengthened element cross-section, the moment of inertia $I_{z}$ of the equivalent cross-section using the Steiner theorem can be calculated using the formula:

(3) $$I_{z}=\frac{ba^{3}}{3}+\frac{bn^{\prime }h_{t}^{3}}{3}+bnt_{1}\left[\frac{t_{1}^{2}}{12}+{\left(c-d_{1}\right)}^{2}\right]+bn^{\prime }t_{1}\left[\frac{t_{1}^{2}}{12}+{\left(c-\frac{t_{1}}{2}\right)}^{2}\right],$$

where:

(4) $$h_{t}=h-a-t_{1}-t_{f},$$

(5) c=h−a,

For the so determined equivalent moment of inertia, the deflection is calculated using the known formulas from the theory of elasticity, depending on the load scheme, taking into account the equivalent moment of inertia and the mean modulus of elasticity in bending $E_{0,mean}$ and taking into account rheological factors.

In the proposed method, the load-bearing capacity of the strengthened cross-section is determined assuming a rectangular distribution of bending stresses in the beam compression zone, disregarding the tensile stresses of wood.

The load-bearing capacity is determined by the exceeding of the load capacity (in terms of elastic behavior) of the compression zone of wood or the load-bearing capacity of the tape, i.e., in determining the load-bearing capacity, the wood in the tension fibers’ area is ignored. It is assumed that the tensile stresses are transferred by the CFRP tape, assuming that a significant wood defect in the tension zone is present.

For such assumptions, the allowable moment resistance of the cross-section is equal to:

(6) $$M_{r}=F_{c}z^{\prime },$$

where:

(7) $$F_{c}=\sigma_{m}0.5ab,$$

(8) $$z^{\prime }=h-\frac{a}{3}-d_{1},$$

$\sigma_{m}$—bending stresses determined based on comparative models,

$\sigma_{m}=f_{m,d}$—design strength of timber in bending, in the case of design calculations.

The tensile stresses in the FRP tape can be determined according to the formula:

(9) $$\sigma_{f}=\frac{M_{r}\left(g+\frac{t_{f}}{2}\right)n}{I_{z}}.$$

Accordingly, for the purpose of comparative verification, for the mean values of the maximum bending moments obtained in the tests for the non-strengthened models of the NWC1 and ZD-2.4 series, bending stresses $\sigma_{m}$ were determined, respectively 42.1 MPa and 39.02 MPa. Then, in accordance with the formula ($M_{r}$), the maximum moment resistance of the cross-section of the strengthened models $M_{r}$ was determined and the obtained $M_{r}$ values were compared with the value of the maximum mean moments M_max_ obtained in the tests for the strengthened models. The results are presented in Table 3. A satisfactory convergence of results was obtained.

## 5. Conclusions

Based on the results of studies carried out in two different laboratories, the following main conclusions can be drawn:

- For the models tested here, an increase in the bending strength of the reinforced beams was observed. The increase in the load capacity of beams with natural wood defects was due to the use of CFRP materials in the tension zone, which increased the ductility of the wood. The load-bearing capacity of the reinforced and glued beams increased by about 23% compared to the control beams, and about 28% for the solid timber beams. Due to the continuity of the tension zone in beams containing defects and reinforced with CFRP materials, an increase in bending stiffness of 36.29% was observed for the glued beams, and about 20% for the solid wood beams. It should be emphasized that the quantitative increases in stiffness and load capacity depend directly on the cross-sectional dimensions and the percentage of reinforcement.
- The applied reinforcement method has a favorable effect on structural elements with reduced mechanical properties in the tension zones. In the case of solid timber and glued timber, there are natural defects in the structure, such as knots, twisted fibers, or others, which have a negative impact, especially in the tension zones, causing discontinuity in this zone, both in bent and tension elements. Due to their high tensile stiffness, CFRP materials effectively reduce the impact of wood weakening caused by e.g., knots, which has a direct impact on the safety of the structure.
- In the course of the above-mentioned research, it was observed that in reinforced beams, in the case of a wood defect in the lower part of the bending beams, these defects did not significantly affect the stiffness of the beams, and the reserve capacity of the tapes allowed them to absorb and safely transfer additional stresses, which effectively improved the safety of the beams.
- The proposed analytical approach seems to show a satisfactory convergence with the results obtained from laboratory tests, but nevertheless it still requires further comparison analyses with the other tests.
- Due to the limited number of studies described in the literature, further research should be continued on the reinforcement of non-homogeneous glued and solid beams with FRP materials, and the impact of structural and geometric characteristics of wood (including wood defects) on the effect of strengthening these beams or checking the behavior of reinforced beams composite materials at the time of their destruction.

## Figures and Tables

**Table 1:** Magnitude of the destructive force and corresponding bending moment for each model.

Model	F_max,(mean)_ [kN]	M_max_ [kNm]
NWC1-1	30.40	15.20
NWC1-2	32.82	16.41
NWC1-3	27.48	13.74
Mean	30.23	15.12
Standard deviation	2.67	1.34
WC2-1	37.96	18.98
WC2-2	38.30	19.15
WC2-3	33.44	16.72
Mean	36.57	18.28
Standard deviation	2.71	1.36
WC3-1	38.04	19.02
WC3-2	37.70	18.85
WC3-3	34.24	17.12
Mean	36.66	18.33
Standard deviation	2.10	1.05
WC4-1	40.04	20.02
WC4-2	38.12	19.06
WC4-3	37.98	18.99
Mean	38.71	19.36
Standard deviation	1.15	0.58

**Table 2:** Magnitude of the destructive force and corresponding bending moment for each model.

Model	F_max,(mean)_ [kN]	M_max_ [kNm]
ZD-2.4/1	35.03	14.01
ZD-2.4/2	57.66	23.06
ZD-2.4/3	57.41	22.96
ZD-2.4/4	49.73	16.89
Mean	49.96	19.98
Standard deviation	10.61	4.24
ZW-2.4S/1	65.79	26.32
ZW-2.4S/2	59.95	23.98
ZW-2.4S/3	57.60	23.04
ZW-2.4S/4	72.65	29.06
Mean	64.00	25.60
Standard deviation	6.72	2.69
ZW-2.4H/1	60.32	24.13
ZW-2.4H/2	51.20	20.48
ZW-2.4H/3	82.02	32.81
Mean	64.51	25.81
Standard deviation	15.83	6.33

**Table 3:** Comparison of experimental and theoretical analysis.

Model	M_max_ [kNm]	M_r_ [kNm]
WC2 (Mean)	18.28	18.51
WC3 (Mean)	18.33	19.40
WC4(Mean)	19.36	20.03
ZW-2.4S (Mean)	25.60	25.23
ZW-2.4H (Mean)	25.81	25.68

## References

[1] Glišović I., Stevanović B., Petrović M. (2015). Bending behaviour of glulam beams reinforced with carbon FRP plates. J. Civ. Eng. Manag..

[2] Jacob J., Barragan O.L.G. (2010). Flexural Strengthening of Glued Laminated Timber Beams with Steel and Carbon Fiber Reinforced Polymers. Master’s Thesis.

[3] Juvandes L.F.P., Barbosa R.M.T. (2012). Bond analysis of timber structures strengthened with FRP systems. Strain.

[4] Wdowiak A., Brol J. (2019). Effectiveness of Reinforcing Bent Non-Uniform Pre-Stressed Glulam Beams with Basalt Fibre Reinforced Polymers Rods. Materials.

[5] Brol J., Wdowiak-Postulak A. (2019). Old Timber Reinforcement with FRP. Materials.

[6] Borri A., Corradi M., Speranzini E. (2013). Bending Tests on Natural Fiber Reinforced Fir Wooden Elements. Adv. Mat. Res..

[7] Nadir Y., Nagarajan P., Ameen M., Muhammed A.M. (2016). Flexural stiffness and strength enhancement of horizontally glued laminated wood beams with GFRP and CFRP composite sheets. Constr. Build. Mater..

[8] Li Y.F., Xie Y.M., Tsai M.J. (2009). Enhancement of the flexural performance of retrofitted wood beams using CFRP composite sheets. Constr. Build. Mater..

[9] Borri A., Corradi M., Grazini A. (2005). A method for flexural reinforcement of old wood beams with CFRP materials. Compos. Part B Eng..

[10] Corradi M., Borri A. (2007). Fir and chestnut timber beams reinforced with GFRP pultruded elements. Compos. Part B Eng..

[11] Johnsson H., Blanksvärd T., Carolin A. (2006). Glulam members strengthened by carbon fibre reinforcement. Mater. Struct..

[12] Plevris N., Triantafillou T.C. (1992). FRP Reinforced Wood as Structural Material. J. Mater. Civ. Eng. ASCE.

[13] Schober K.U., Rautenstrauch K. (2005). Experimental investigations on flexural strengthening of timber structures with CFRP. International Symposium on Bond Behaviour of FRP in Structures (BBFS 2005).

[14] Schober K.U., Rautenstrauch K. (2007). Post-strengthening of timber structures with CFRP’s. Mater. Struct..

[15] Jankowski L.J., Jasieńko J., Nowak T.P. (2010). Experimental assessment of CFRP reinforced wooden beams by 4-point bending tests and photoelastic coating technique. Mater. Struct..

[16] Meier U. (1995). Strengthening of structures using carbon fibre/epoxy composites. Constr. Build. Mater..

[17] Parisi M.A., Piazza M. Rehabilitation of timber structures by new materials and connectors. Proceedings of the 10th International Conference Structural Faults & Repair 2003—Extending the Life of Bridges, Concrete & Composites, Buildings, Masonry & Civil Structures’.

[18] Johns K.C., Lacroix S. (2000). Composite reinforcement of timber in bending. Can. J. Civ. Eng..

[19] Triantafillou T.C. (1997). Shear reinforcement of wood using FRP materials. J. Mater. Civ. Eng. ASCE.

[20] Gentile C., Svecova D., Saltzberg W., Rizkalla S.H. Strengthening for Timber Bridges. Proceedings of the World Wise’99.

[21] Brol J., Wdowiak A. (2017). The Use of Glass and Aramid Fibres for the Strengthening of Timber Structures.

[22] Brol J., Nowak T., Wdowiak A. (2018). Numerical Analysis and Modelling of Timber Elements Strengthened with FRP Materials.

[23] Wdowiak A. (2016). Analysis of bent timber beam reinforcement with the application of composite materials. Struct. Environ..

[24] Wdowiak A. (2019). Właściwości Strukturalno—Wytrzymałościowe Zginanych Belek Drewnianych Wzmocnionych Kompozytami Włóknistymi [Structural and Strength Properties of Bent Wooden Beams Reinforced with Fibre Composites]. Ph.D. Thesis.

[25] Wdowiak A., Kroner A. (2017). Wpływ niejednorodności struktury zginanych belek z drewna klejonego na efekt ich wzmocnienia. Mater. Bud..

[26] Wdowiak A., Brol J. (2019). Methods of strength grading of structural timber—Comparative analysis of visual and machine grading on the example of Scots pine timber from four natural forest regions of Poland. Struct. Environ..

[27] Fiorelli J., Dias A.A. Evaluation of the Structural Behavior of Wood Beams Reinforced with FRP″. Proceedings of the 7th World Conference on Timber Engineering, WCTE 2002, Perpustakaan Negara Malaysia.

[28] Buell T.W., Saadatmanesh H. (2005). Strengthening Timber Bridge Beams Using Carbon Fibre. J. Struct. Eng..

[29] Alam P., Ansell M.P., Smedley D. (2009). Mechanical Repair of Timber Beams Fractured in Flexure Using Bonded-in Reinforcements. Compos. B Eng..

[30] Brol J. (2005). Analiza Doświadczalno-Teoretyczna Wzmacniania Konstrukcji Drewnianych Kompozytami Polimerowo-Węglowymi [Experimental and Theoretical Analysis of Wooden Structures Strengthening with Polymer-Carbon Composites]. Ph.D. Thesis.

[31] Nowak T. (2005). Analiza Pracy Statycznej Zginanych Belek Drewnianych Wzmacnianych Przy Użyciu CFRP [Analysis of the Static Work of Bent Wooden Beams Reinforced with CFRP]. Ph.D. Thesis.

[32] Mukhopadhyaya T., Naskarb S., Deyc S., Chakrabartid A. (2019). Condition assessment and strengthening of aged structures: Perspectives based on a critical case study. Pract. Period. Struct. Des. Constr..

[33] (2004). Moisture Content of a Piece of Sawn Timber—Part 2: Estimation by Electrical Resistance Method.

[34] (2007). Moisture Content of a Piece of Sawn Timber—Part 3: Estimation by Capacitance Method.

[35] (2013). Coniferous Construction Timber Sorted by Strength Methods.

[36] (2012). Timber Structures—Structural Timber and Glued Laminated Timber—Determination of Some Physical and Mechanical Properties.

[37] (2010). Design of Timber Structures—Part 1-1: General—Common Rules and Rules for Buildings.

